# Spontaneous Ligand Access Events to Membrane-Bound Cytochrome P450 2D6 Sampled at Atomic Resolution

**DOI:** 10.1038/s41598-019-52681-w

**Published:** 2019-11-11

**Authors:** André Fischer, Martin Smieško

**Affiliations:** University of Basel, Department of Pharmaceutical Sciences, Basel, 4056 Switzerland

**Keywords:** Computational chemistry, Molecular modelling

## Abstract

The membrane-anchored enzyme Cytochrome P450 2D6 (CYP2D6) is involved in the metabolism of around 25% of marketed drugs and its metabolic performance shows a high interindividual variation. While it was suggested that ligands access the buried active site of the enzyme from the membrane, no proof from unbiased simulations has been provided to support this hypothesis. Laboratory experiments fail to capture the access process which is suspected to influence binding kinetics. Here, we applied unbiased molecular dynamics (MD) simulations to investigate the access of ligands to wild-type CYP2D6, as well as the allelic variant CYP2D6*53. In multiple simulations, substrates accessed the active site of the enzyme from the protein-membrane interface to ultimately adopt a conformation that would allow a metabolic reaction. We propose the necessary steps for ligand access and the results suggest that the increased metabolic activity of CYP2D6*53 might be caused by a facilitated ligand uptake.

## Introduction

Cytochrome P450 enzymes (CYPs) are essential proteins involved in the detoxification of foreign compounds reaching the human body. CYP2D6 accounts for the oxidative metabolism of roughly 25% of all marketed drugs and therefore belongs to the most relevant enzymes involved in phase I biotransformation. In addition, the enzyme is subject to a high interindividual variation in metabolic performance due to a genetic polymorphism. In drug therapy, this can ultimately lead to either severe adverse effects or the suppression of a therapeutic effect^1^. The allelic variant CYP2D6*53, which harbors the two amino acid mutations F120I and A122S, shows an increased metabolic rate towards several substrates in experiments indicating a pending designation as ultrarapid metabolizer (UM) phenotype^2–7^.

The active site of CYPs is located in a buried cavity inside the enzyme that is connected to the surrounding environment by tunnels^2,8–14^. These tunnels are believed to influence both the poorly understood substrate specificity and binding kinetics of CYPs^1,11,13,15^. As the prediction of CYP metabolism is of major importance for drug development^1^, the influence of enzyme tunnels on small molecule binding has been intensively investigated^2,10,13,16,17^. Available experimental methods have only limited applicability for the determination and characterization of enzyme tunnels or complex transport phenomena. While crystal structures only provide a static view of the protein and fail to capture dynamic events, techniques such as NMR spectroscopy can produce dynamic information on the conformations of a protein^8,9,16^. Nonetheless, atomic details on the dynamic uptake of CYP2D6 ligands has not yet been produced by any experimental method^16^. Computer simulations, on the other hand, have provided fundamental insight into the transport process of ligands in CYPs. Various molecular dynamics (MD) simulation techniques have been applied to study the dynamic tunnels and their capability to transport ligands in CYPs^10,11,15,16,18,19^. While most groups focused on the egress routes of ligands from the active site, only a handful of studies were focused on access routes^10,18,19^ which are likely to be different^13,20^. Due to the long timescale of such molecular processes^21^, biasing potentials have been applied in nearly all studies to increase the likeliness of a successful translocation. Although two studies applied unbiased MD protocols to study the access to a CYP, they were focused on a soluble, bacterial enzyme^12^. Studies with CYP2D6 were limited to the determination and characterization of enzyme tunnels independent of a particular ligand^2,4,17,22^. Overall, the access of ligands to mammalian CYPs is poorly understood and could not yet be observed in its full complexity in unbiased simulations.

In contrast to prokaryotes, mammalian drug-metabolizing CYPs are membrane-anchored and their globular domain is partially embedded in the membrane^14^. Based on the spatial location of several tunnels at the protein-membrane interface and the rather lipophilic character of many CYP ligands, it was suggested that ligands may access the active site from the membrane compartment and leave it efficiently through solvent-facing tunnels^2,8,10,13,14,18,20^. For example, it was shown that the preferred position of ibuprofen relative to a membrane agrees with superficial entry points of access tunnels in CYP2C9^13^. In another study, the spontaneous, non-reproducible insertion of a membrane lipid in an enzyme tunnel was observed^10^. No unbiased MD protocol was applied to confirm this hypothesis in a mammalian CYP, let alone in CYP2D6.

In this study, we performed over 20 *μ*s of unbiased MD simulations with the aim to study the access of ligands to the buried active site of CYP2D6 in a model of the full-length structure of the enzyme anchored and partly embedded in a biological membrane^2^. In eight simulations, we observed substrates accessing the buried active site cavity of the enzyme via specific tunnels located at the protein-membrane interface. We propose the key steps governing a successful access of the ligand. Further, the results support the pending designation of the allelic variant CYP2D6*53 as a cause for ultrarapid metabolizer phenotype based on a more efficient ligand uptake compared to the wild-type.

## Results and Discussion

### Access of CYP2D6 ligands from the protein-membrane interface

**Figure 1:**
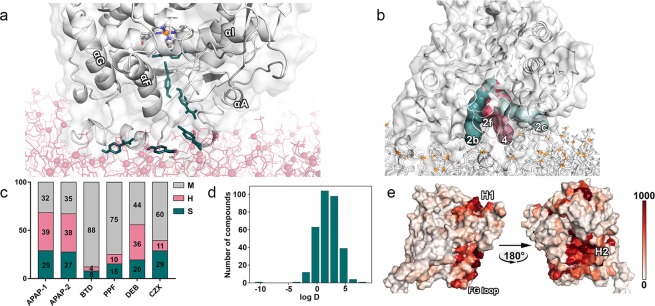
Access tunnels and the spatial preference of ligands. (**a**) APAP accessing CYP2D6 from the protein-membrane interface in simulation #3. The ligand, shown in pine green, is starting outside the enzyme (bottom left) to access the active site. The membrane is colored red and phosphorus atoms are shown in sphere representation. Four helices are indicated for better orientation. (**b**) The four largest access tunnels are shown with the structure of CYP2D6*53. For orientation, the membrane phosphorus atoms are shown in orange. Other tunnels facing the solvent are not shown for simplicity. (**c**) The averaged distribution of ligands relative to three compartments consisting of membrane (M), head groups (H), and the remaining space (S) is shown. The two different measurements for APAP resulted from simulations at different temperatures. (**d**) Plot of the log D values predicted for a database of CYP2D6 ligands. The plot was generated using Matplotlib^47^. (**e**) A visualization of the hotspots of APAP on the surface of CYP2D6 is shown in different shades of red. The scale from 0–1000 describes the cumulative number of ligand heavy atoms in a 5 Å radius of the CB atom (CA atom for glycine) of the protein amino acids. Two large hotspots were denoted as H1 and H2. For better orientation, the position of the FG loop is indicated.

Due to recent advances in computational capabilities, researchers are able to observe rare molecular events, such as intramolecular diffusion, inaccessible to laboratory experiments, using computer simulations^16,20,21,23^. Simulations of events such as ligand binding can not only improve our understanding of fundamental molecular processes, but can also be used to estimate binding affinities and residence times of drug candidates^24^. Specifically for CYPs it was shown that distinct tunnels, predominantly located at the protein-membrane interface, connect the buried active site to its surrounding environment. Together with the general hydrophobicity of CYP substrates, this led to the widely discussed hypothesis of ligand access from the membrane^2,8,10,13,14,18,20^. Although, this hypothesis could not yet be proven based on a complete, unbiased trajectory of a ligand accessing the active site, it was supported by a study applying accelerated MD simulations to CYP3A4. However, the used technique might not have accounted for the exact dynamics of the system due to the biasing potential^25^. Further, the accordance of other simulation techniques involving biasing potentials to conventional MD is not inherently given as it was shown for adaptive sampling methods^26^. Therefore, unbiased simulations could serve as a blueprint to validate accelerated simulations and other biased simulation techniques that can then be used to tackle complex issues such as the determination of drug binding affinities. Previously, unbiased simulations were limited to the soluble, bacterial Camphor 5-monooxygenase (CYP101A1) meaning that the involvement of the membrane could not be considered^12,27^. Here, we conducted unbiased MD simulations to investigate the access of ligands to CYP2D6 in a membrane-anchored model. We were able, for the first time, to observe the complete translocation of a ligand from the solvent to the buried active site cavity of a mammalian CYP in multiple simulations (Table 1 and S3). We randomly distributed 20 ligand molecules of either acetaminophen (APAP), butadiene (BTD), chlorzoxazone (CZX), debrisoquine (DEB), or propofol (PPF) inside the aqueous phase of the periodic boundary systems in an average distance of 13.8 Å (ranging between 2.7 and 49.2 Å) to the next protein heavy atom (Fig. S1 and Table S4). In two exploratory simulations, a smaller number of ligands was used. From the solvent, the accessing ligands APAP and BTD sampled the simulation system, adhered to the tunnel entrance, and translocated to the active site of the allelic variant CYP2D6*53 through membrane-facing tunnels (Figs 1a,b and S3). Remarkably, our simulation setup did not require prior knowledge of the binding path or the location of the active site and produced ten access events in a total of 24 simulations.

**Table 1:** For each access event, the simulation identifier, the used protein structure, the accessing ligand, the time it took to be recognized at the tunnel entrance (T_R_), the time it took for translocation to the active site (T_T_), and the tunnel it translocated through, is shown.

Simulation	Structure	Ligand	T_R_ (*μ*s)	T_T_ (*μ*s)	Tunnel	SOM
#3	CYP2D6*53	APAP-18	0.04	0.28	2f	yes
#4	CYP2D6*53	APAP-7	0.35	0.19	2f	yes
		APAP-18	n/a	n/a	2f	no
#5	CYP2D6*53	APAP-18	0.15	0.82	4	yes
#6	CYP2D6*53	APAP-6	0.61	0.08	2b	yes
#7	CYP2D6*53	APAP-3	0.27	0.19	2b	yes
		APAP-8	0.61	0.41	2b	yes
#8	wild-type	APAP-20	1.42	n/a	2f	no
#13	CYP2D6*53	BTD-11	0.03	0.03	2c	yes
#14	CYP2D6*53	BTD-3	0.01	0.03	2c	yes

Further it is indicated if the ligand reached a pose placing its SOM in a conformation agreeing with a metabolic reaction.

In two simulations, we observed two ligands entering the active site consecutively. Notably, we observed only one, with 1.42 *μ*s needed for recognition comparatively slow, access event with the wild-type structure despite substantial simulation efforts. Interestingly, the accessing BTD molecules were quickly recognized at the tunnel entrance without prior contact to the membrane (Movie S1). The other simulations performed in the context of this study did not result in the successful access of a ligand to the active site. Potentially, the simulations with DEB, PPF, and CZX could have led to ligand binding events if prolonged appropriately, but due to the high computational cost of the simulations we focused on the most promising candidate to reproducibly simulate binding in the given timescale, which was APAP based on the analysis of the first 480 ns of the simulations. The in-depth analysis of the access events revealed a considerable heterogeneity among them regarding the time taken to access the enzyme, the favored tunnel, as well as the adopted conformation in the active site (Table 1). After a slow recognition phase, the ligands adhered to one of the tunnel entrances located close to the protein-membrane interface. In two simulations (#3 and #5), the starting position was relatively close to the tunnel entrance leading to a fast recognition phase and limited sampling of the complete simulation system (Table 1). Further on, simulation #4 was a replica simulation meaning that one of the two accessing ligands (APAP-18) already started at the tunnel entrance, while the second accessing ligand (APAP-7) started in over 50 Å distance from the next protein heavy atom. In the remaining simulations, we could observe the recognition phase to be the rate-limiting step of the process with extensive sampling of the simulation system. An initial phase to recognize the tunnel entrance followed by a temporary superficial association, as we observed it, stands in agreement with a model describing a two-step binding process allowing kinetically efficient ligand uptake. This would enable the ligand to efficiently minimize the, otherwise even longer, recognition phase and is in accordance with recent observations regarding proteins with similarly buried active sites such as CYP101A1 and nuclear receptors^11,21,27–30^. In the active site, eight out of ten accessing ligands adopted a pose which would allow an oxidation reaction to proceed at a site of metabolism (SOM) that would ultimately result in a metabolite in agreement with experiment^31^. In simulation #4, two ligands occupied the binding site simultaneously and one molecule was accommodated distant (>10 Å) from the heme. Further, simulation #8 with the wild-type structure did not result in a pose that was in agreement with metabolism of APAP.

### Validation of the simulations

The models used in this study originated from our previously built, characterized, and validated full-length models of wild-type CYP2D6 as well as the allelic variant CYP2D6*53^2^. Here, we evaluated key parameters and proved their accordance to our previous observations and experimentally derived literature values (Fig. S3 and Table S5). These parameters included the burying depth of the enzyme, the heme tilt angle that describes the orientation of the enzyme to the membrane, as well as the root mean square deviation (RMSD) and root mean square fluctuation (RMSF). As mentioned above, we distributed multiple ligands into one simulation system. In order to determine to which degree ligands interacted with each other, we examined the presence of ligands in the proximity of the accessing molecule. Overall, we detected few interactions to other ligands, with the clear exception in the case of a double access event, where contacts were expected (Table S6). To rule out that large agglomerates formed during the simulations, we measured the distances of all ligands in the simulations and averaged them for each MD frame. While the data indicates that there was no large formation of agglomerates, the fluctuations of the average distance indicated the formation of transient small agglomerates (Fig. S4). In comparison, the simulations with PPF showed a slightly increased trend for agglomeration, which might have contributed to the fact, that we did not observe any PPF molecules entering a tunnel. For a more detailed description of the validation, please refer to SI Results and Discussion.

### Preference of ligands for protein, tunnels, and membrane

Regarding the difference in the preferred tunnels for translocation, it was suggested that multiple tunnels might serve as an access route to CYPs, specifically to govern the substrate specificity of the enzyme^8,20^. In particular, tunnel entrances differing in burying depth within the membrane would allow the uptake of ligands varying in lipophilicity and therefore in their favored position relative to the membrane^13,14,18^. Indeed, the environment around the entrances of tunnels that were favored by the ligands varied as it can be seen at the example of tunnel 2c (Fig. 1b). Furthermore, our analysis of the favored position relative to the membrane revealed significant differences among CYP2D6 ligands (Fig. 1c), supporting this presumption. We logically divided the simulation box into three zones consisting of the membrane core (M), the head group region (H), and the remaining space made up of protein and solvent (S). BTD, CZX as well as PPF mainly partitioned towards the membrane core, while APAP preferred the head group region. The two slightly different temperatures in the simulations with APAP only had minor impact on the distribution. Despite the relatively similar behavior of DEB and APAP, we did not observe any DEB molecules accessing the enzyme. This might have been caused by the bulkier character of DEB requiring larger conformational changes for uptake as well as its slightly greater preference for the membrane core. During MD simulations, long residence times in the membrane core potentially reduce the probability to observe ligand access in the microsecond timescale. This is supported by the fact that both accessing lipophilic BTD molecules quickly entered the enzyme through the mostly solvated entrance of tunnel 2c near the protein-membrane interface without thorough sampling of the membrane core in our simulations. Likely, the hydrophobic milieu inside tunnel 2c^2^ offered a favorable environment for the accessing BTD molecules. The calculated distribution coefficients (log D), describing the general preference of ligands towards a hydrophobic environment, revealed a peak around 2.5 for CYP2D6 ligands (Fig. 1d). The clear difference in lipophilicity between APAP and BTD potentially influenced the selected tunnel for translocation to the active site. While APAP did not show a clear preference for a specific tunnel, BTD translocated through tunnel 2c in both access events (Table 1), which is likely associated with the relatively high lipophilicity of the amino acid residues lining this tunnel^2^. This, together with their different positions relative to the membrane compartment, underlines the importance of tunnels and their constitution for substrate specificity, since distinct chemical and geometrical features allow selective uptake of substrates^11,13,14^.

Only recently, ligand-dependent long-range motions have been detected in an allosteric mechanism for CYP101A1, in which the occupancy of a peripheral site on the enzyme surface induces the opening of an access tunnel^12^. We identified two main sites (denoted as H1 and H2) during the nine simulations with APAP included in this analysis. Site H1 corresponded to a pocket around helices C, E, and H similar to the described allosteric site in CYP101A1, while site H2 highlighted a broad surface around helices F and A as well as the *β* sheet 4 close to the entrance of tunnel 2f (Fig. 1e). The H1 site was distant from the opening of any of the major tunnels. We determined these sites on the protein surface according to the number of ligand heavy atoms that were present in a 5 Å sphere around the amino acid residues in the respective simulations. Although we frequently observed the occupancy of the described H1 site in our simulations with APAP, the data indicated a secondary role of the above-mentioned allosteric mechanism for CYP2D6, since H1 occupancy was not mandatory for a successful translocation (Table S7). However, the data indicated that the H1 site might be involved in the opening of tunnel 2f. In this context, it was shown that the association of redox partners and dioxygen binding might additionally influence the conformational state of the enzyme^32^. Since the H2 site corresponds to a surface near the entrance of tunnel 2f, the data additionally supports the above-mentioned two-step binding mechanism. The BTD molecules did not sample the protein surface as extensively as APAP (Fig. S5).

### Structural adaptation of the protein

**Figure 2:**
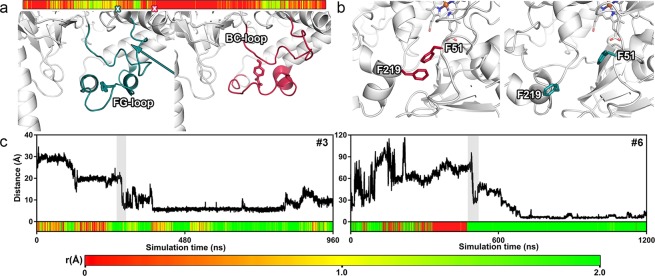
Opening and adaptation of ligand tunnels. (**a**) APAP is shown at the entrance of tunnel 4 (defined by the FG loop) in simulation #5 at two different time points. On the left side the FG loop is clearly extended, while it presented a different conformation after the ligand advanced (right). The simultaneous movement of the BC loop lead to the reversible narrowing of tunnel 2c (arrow, left side), as it is indicated by the time-evolved bottleneck radius (shown above) of the simulation. The respective frames are marked on the color bar. (**b**) Gate between F51 and F219 shown in two different states. While the gate is closed at the beginning of simulation #3 (left), it adopted an open state after ligand translocation in simulation #4, forming a so-called wing gate^9^. (**c**) The distance between the SOM and the heme iron is plotted against the simulation time as well as the time-evolved bottleneck radius. The simulation identifier is shown at the top right of the plots, while gray bars indicate the period of tunnel translocation. The legend at the bottom indicates the coloring scheme for the bottleneck radii.

**Figure 3:**
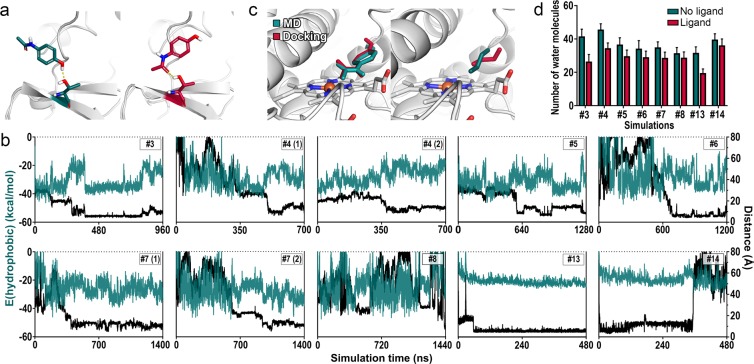
Driving force for translocation, poses in the active site, and its desolvation. (**a**) T394 acting as a guiding rail for the ligand by hydrogen bonding. (**b**) The hydrophobic energy was plotted against the distance between the ligand SOM and the heme iron as well as the simulation time for all simulations with a successful access event. (**c**) Comparison of best matching poses of APAP (left) and BTD (right) obtained from MD simulations and molecular docking. (**d**) The number of water molecules in the active site in presence and absence of a ligand.

Since crystal structures do not provide a comprehensive explanation on how ligands access or leave the buried active site of CYPs, the protein has to undergo structural fluctuations to allow ligand access^8,9,15,28^. Indeed, we detected several dynamic adaptations of the protein that were, in certain cases, directly related to the accessing ligand. Similar to a recent study^27^, the rearrangements did not alter the overall structural composition of the enzyme. The conformational changes of the secondary structure were mostly located on the protein surface, predominantly in regions with increased flexibility, and sometimes even in great distance from the ligand. In simulation #6 for example, the ligand induced a reversible conformational change of the FG loop, forming the entrance of tunnel 4, in order to propagate. This rearrangement additionally impacted the nearby BC loop leading to the tightening of tunnel 2c formed by this loop, as it was reflected by its temporarily decreased bottleneck radius (Fig. 2a). This points towards an induced-fit mechanism as opposed to conformational selection^33^. Only recently the latter was proposed to be the main mechanism for multiple CYPs including CYP2D6^30^, even though for several other CYPs induced-fit scenarios were not ruled out. Besides movements of the FG loop, we observed helix A, the BC loop, the HI loop, and helix B to be involved in conformational changes (Table S8). Since the mentioned structural adaptations often occurred in tunnels during the translocation of the ligand, it is likely that those structural elements are involved in gating the active site, as it was shown for other enzymes^9,32^. On the level of amino acids, gates frequently consist of aromatic residues^2,9,11,32^. We found individual residues to be involved in the gating of tunnel 2f, where F51 and F219 showed different conformations before and after ligand translocation (Fig. 2b) without direct involvement of the ligand. In contrast to the above-mentioned conformational changes at tunnel 4, the opening of this gate can be best described as a conformational selection mechanism since the conformational change took place independent of the ligand molecule^33^. This suggests that depending on the tunnel used for translocation both induced-fit and conformational selection can describe the observed conformational changes. F51 and F219, among several other residues, functioned as bottleneck residues (Fig. S6) which are often involved in gating tunnels^9^. Gates regulating enzyme tunnels are typically located at their most narrow part, which is determined by the bottleneck radius, and can be formed by secondary structural elements or individual residues. To determine the opening degree of the tunnels used for translocation, we monitored their bottleneck radii in simulations with access events (Figs 2c and S7). Based on the bottleneck radius, we discovered the favored tunnels to be open during the translocation of the ligand. Especially in simulation #6, it is clearly visible that the tunnel was closed when the ligand was approaching and opened shortly before its translocation (Fig. 2c). Even though simulation #13 with BTD presented a similar opening pattern, the tunnel closed after translocation, implying conformational adaptations on the side of the protein. The conformational changes in relation to the movement of the ligand are in accordance with recent findings on an induced-fit driven mechanism of ligand binding to CYP101A1^27^. In contrast to commonly described induced-fit mechanisms in active sites^34,35^, the described motions occurred at peripheral sites of the protein, such as the FG loop that is involved in the formation of multiple enzyme tunnels. Interestingly, we also observed motions of secondary structural elements in a significant distance (over 10 Å) from the ligand during the exploration of the active site such as the movements of the HI loop. In general, structural adaptations and protein flexibility are not only important to improve our understanding of the structural mechanism behind ligand access^8^, but are also crucial to be considered in molecular docking calculations^35^. Even though MD-simulations are regularly used in a supporting role to post-process and refine poses obtained from docking, it was recently suggested that docking might even be replaced by MD-based techniques^35,36^. Since our simulations lead from an unbound state to a bound state in the active site, this further supports these suggestions. Additionally, the results from docking APAP and BTD indicated, that the poses generated by flexible docking were strongly dependent on the used receptor structure (Table S9). When we compared poses obtained from docking and MD, we identified a similar (RMSD < 2 Å) pose in three out of eight access simulations (Fig. 3a and Table S10). Our results show that the poses obtained from MD can closely resemble the ones obtained from docking, but they additionally allow to get insight into the dynamic interplay of the protein and the ligand and offer more potential for interpretation. Due to high computational expense that comes with conventional MD simulations as we used them, simulation techniques employing biasing potentials would offer a higher throughput for pose prediction from a completely unbound state^25,36^. Together with the above-mentioned significant structural adaptations of the protein backbone, we conclude a rather limited applicability of traditional docking methods to such flexible proteins and support the use of MD-based methods.

### The driving forces for translocation

Although the opening of gates was crucial for a successful ligand translocation, additional forces are required for the ligand to propagate to the active site in order for metabolism to occur steadily and reproducibly. We analyzed the nature of the interaction between the ligand and the protein in each simulation with an access event to identify the driving force for ligand translocation through the tunnels. Therefore, we considered contributions from electrostatics, hydrophobic contacts as implemented in the VSGB 2.0 model^37^, and hydrogen bonds based on a term that accounts for their directionality^38^. While the ligands showed favorable hydrophobic interactions towards the membrane lipids at first, they generally decreased upon contact to the protein surface and increased again as the ligand advanced through the tunnel to enter the active site (Fig. 3b). The correlation was especially evident at the example of simulation #3, where a slight displacement of the ligand from its favored pose in the active site directly caused a substantial weakening of the hydrophobic energy. Even though not all simulations showed a clear correlation (#4 and #5), most of them presented a trend for a gain in hydrophobic energy during the translocation from the enzyme surface to the buried active site, where the known hydrophobic environment^1,15^ seemed to offer a favorable milieu for the ligands. The relatively fast access of both non-polar BTD molecules added additional evidence for the relevance of hydrophobicity. Contributions from electrostatics and hydrogen bonds were constantly present, but remained steady whether the ligand was in the solvent, membrane, or in the active site (Fig. S8). However, polar contacts allowed ligands to adhere to the tunnel entrance and we observed distinct hydrogen bonds to guide APAP toward the active site by consecutively interacting with different heteroatoms (Figs 3a and S9a,b). This supports the role of polar contacts as a secondary driving force for the access of APAP, while BTD obviously could not form polar contacts due to the lack of heteroatoms. Together with gates, distinct polar interactions in enzyme tunnels have to play a relevant role in regulating the substrate specificity of the enzyme since hydrophobicity is a general property shared by many CYP substrates. The residues that interacted with the ligands during recognition, translocation, and the phase in the active site are shown in Table S11.

Other factors potentially influencing ligand uptake include the desolvation of the active site and the ligand. The displacement of water molecules in a binding site is a common strategy to optimize the binding affinity of compounds in the field of medicinal chemistry. Depending on the environment of the water molecule, the displacement can be both favorable or unfavorable^39,40^. We identified a trend for a decreased number of water molecules in the active site when a ligand occupied it (Fig. 3d) indicating a modest desolvation effect. However, the absolute numbers of displaced water molecules did not converge, likely due to the comparably small size of the ligands, the known enlarged active site cavity of CYP2D6*53^2^, and the overall heterogeneity of the individual access events. Similarly, the number of water molecules forming the hydration shell around the ligand did show great variation with no clear trend for APAP (Fig. S9c,d). On the other hand, the number of water molecules accompanying the hydrophobic BTD molecules decreased to a significant amount on their journey from the bulk solvent to the active site. The desolvation of a drug-like ligand, associated with its binding to hydrophobic active sites^41^, is generally a penalizing contribution toward affinity. In the case of BTD however, the solvation energy presents a positive value (0.61 kcal/mol)^42^, leading to a favorable contribution for its desolvation. This suggests the partial desolvation of BTD as a favorable contribution toward its translocation. The, in this case, negligible influence of the conformational flexibility (Fig. S10) on the access process is discussed in the SI Results and Discussion.

### Increased metabolic activity of CYP2D6*53

Measurements of the enzymatic activity of allelic variant CYP2D6*53 have revealed increased metabolic rates towards bufuralol, dextromethorphan, and N-desmethyltamoxifen^3,6,7^. In contrast, a recent study reported a decrease in the clearance of primaquine^43^. It is suspected that the mostly increased metabolic rates of CYP2D6*53 are caused by an enlargement of enzyme tunnels allowing efficient ligand uptake to the enzyme^2,4^. Altogether, this resulted in the speculation of an ultrarapid metabolizer (UM) phenotype for this allelic variant, which is usually only granted to phenotypes resulting from gene duplication^3,4,7^. Similar to our previous observations, tunnel 2b had a wider average bottleneck radius in CYP2D6*53 compared to the wild-type, likely due to the F120I mutation located at the entrance to the active site^2^. Further, the ligand access was faster in the CYP2D6*53 variant compared to the wild-type (Table 1). Therefore, our results moderately support the potential designation of CYP2D6*53 as UM phenotype based on a more efficient ligand uptake of the analyzed substrates.

## Conclusion

The results presented here revealed the atomic mechanism of ligand uptake to the buried active site of membrane-anchored CYP2D6 from the protein-membrane interface. The ligands APAP and BTD accessed the enzyme via different enzyme tunnels, which supports the notion of multiple functional tunnels within a single protein system. The tunnels varied in their burying depth in the membrane which would allow ligands differing in lipophilicity to access the active site. However, presumably due to the relative bulkiness of DEB, CZX and PPF and their increased partitioning towards the membrane core, the simulations with these ligands did not result in any binding events in this timescale. We show that the access process is linked to conformational adaptations of the protein backbone that can occur either in close proximity or in significant distance from the ligand molecule. While the conformational change at tunnel 4 followed an induced-fit mechanism, we also observed motions of residues that could be better described by a conformational selection model suggesting that both processes can occur in CYP2D6 depending on the tunnel. Together with the fact, that our simulations lead from an unbound to a bound state in a fully flexible unbiased manner, we support the use of MD-based techniques as opposed to docking, which stands in accordance with recent suggestions in the literature. Further, we show that the uptake process is mainly driven by hydrophobic interactions with a secondary role for polar contacts during recognition and translocation of the ligand molecules. In addition, the binding process was potentially facilitated by a modest desolvation of the active site. The difference in burying depth, physicochemical properties, and geometrical features of the tunnels influence their capability to transport certain ligands and therefore likely influence the specificity of the enzyme. Similarly, our results indicate that the increased metabolic rates of the allelic variant CYP2D6*53 might be caused by an efficient uptake of ligands compared to the wild-type enzyme. Our study could serve as a blueprint for simulations employing biasing potentials and it proves the capability of unbiased MD simulations to study ligand transport processes.

## Methods

As a starting point for our simulations we used our previously validated full-length model of wild-type CYP2D6 and CYP2D6*53 anchored to a membrane^2^. After randomly distributing multiple substrates in the solvent space around the enzyme, we performed over 20 *μ*s of total unbiased MD simulations with multiple replica systems and various ligands with the aim to study ligand partitioning and to observe a translocation from the bulk solvent to the buried active site of the enzyme. All simulations were performed using the Desmond engine^44^. To determine the enzyme tunnels, we used CAVER 3.0^45^. For the subsequent calculations, we either used workflows included in the Schrodinger Small-Molecule Drug Discovery Suite^46^ or in-house routines. For a complete set of detailed materials and methods, please refer to SI Methods.

## Supplementary information

Supplementary Information

## Data Availability

The datasets generated during and/or analysed during the current study are available from the corresponding author on reasonable request. The source code for the described in-house routines will be made publicly available upon publication in our repository at https://github.com/mmodbasel/scripts-001.
