# Supplementary material for: Spontaneous Ligand Access Events to Membrane-Bound Cytochrome P450 2D6 Sampled at Atomic Resolution
Source: Sci Rep. 2019 Nov 11;9:16411. doi: 10.1038/s41598-019-52681-w (PMC6848145; doi:10.1038/s41598-019-52681-w)
Supplement: Supplementary file 1 — Supplementary Information [file 41598_2019_52681_MOESM1_ESM.pdf]

Supplementary Information

Spontaneous Ligand Access Events to Membrane-Bound Cytochrome P450 2D6 Sampled at Atomic Resolution

André Fischer and Martin Smieško*

Abstract: The membrane-anchored enzyme Cytochrome P450 2D6 (CYP2D6) is involved in the metabolism of around 25% of marketed drugs and its metabolic performance shows a high interindividual variation. While it was suggested that ligands access the buried active site of the enzyme from the membrane, no proof from unbiased simulations has been provided to support this hypothesis. Laboratory experiments fail to capture the access process which is suspected to influence binding kinetics. Here, we applied unbiased molecular dynamics (MD) simulations to investigate the access of ligands to wild-type CYP2D6, as well as the allelic variant CYP2D6*53. In multiple simulations, substrates accessed the active site of the enzyme from the protein-membrane interface to ultimately adopt a conformation that would allow a metabolic reaction. We propose the necessary steps for ligand access and the results suggest that the increased metabolic activity of CYP2D6*53 might be caused by a facilitated ligand uptake.

Table of Contents

Computational Methods

Computational setup and general simulation conditions 2
Ligand preparation 2
Ligand access simulations and validation 3
Preference of ligands for protein, tunnels, and membrane 4
Tunnel analysis 5
Ligand-protein and ligand-membrane energies 5
Hydration analyses 5
Docking and pose comparison 5

Supporting Results and Discussion

Access of CYP2D6 ligands from the protein-membrane interface 6
Model validation 7
Preference of ligands for the protein, tunnels, and the membrane 10
Structural adaptation of the protein 11
The driving forces for translocation 14

Supporting Movie 16
Supporting References 17

Computational Methods

Computational setup and general simulation conditions

The molecular dynamics (MD) simulations were performed on consumer-grade desktop computers equipped with graphics processing units (GPUs) or a dedicated, rack-mounted GPU server. On all used machines in this study, the Desmond simulation engine (v2016-4) was installed in a Linux environment[1]. Prior to the MD simulations, the default relaxation protocol of Desmond (Table S1) was conducted.

Table S1. Relaxation protocol prior to MD simulation.

Desmond stage	Procedure
1	Task (reading files, initializing parameters)
2	Simulate, Brownian Dynamics, NVT, T = 10 K, small time steps, and restraints on solute heavy atoms, 100 ps
3	Simulate, NVT, T = 10 K, small time steps, and restraints on solute heavy atoms, 12 ps
4	Simulate, NPT, T = 10 K, and restraints on solute heavy atoms, 12 ps
5	Solvate pocket
6	Simulate, NPT and restraints on solute heavy atoms, 12 ps
7	Simulate, NPT and no restraints, 24 ps

This table was adapted from our previous work[2].

We chose the OPLS_2005 force field in an NPT ensemble and combined the Martyna-Tobias-Klein barostat with a relaxation time of 2.0 ps at 300 K with the Nose-Hoover thermostat at a relaxation time of 1.0 ps. We used the u-series[3] method to treat long-range interactions combined with a cutoff of 9 Å for short range interactions. By default, the M-SHAKE algorithm was used to constrain bonds to hydrogen atoms and no hydrogen mass partitioning was applied. The orthorhombic periodic boundary boxes were solvated with TIP3P water molecules, just as in our previous work[2]. The time step of the RESPA integrator was set to 2.0 fs and frames with atomic coordinates were written every 48 ps in all simulations. If it is not indicated otherwise, figures of molecules were generated using PyMol[4] and plots were generated using Prism GraphPad[5].

Ligand preparation

To perform the ligand access simulations, we selected five ligands, including acetaminophen (APAP), 1,3-butadiene (BTD), chlorzoxazone (CZX), debrisoquine (DEB), and propofol (PPF) from a database of CYP2D6 ligands[6]. We retrieved the two-dimensional (2D) ligand structures from the PubChem structure database (Table S2)[7]. Maestro from the Schrodinger Small-Molecule Drug Discovery Suite[8] provides the Epik[9] environment to predict the protonation state of ligands. After pipelining all ligands through Epik at physiological pH (7.4), with water as solvent, and the inclusion of tautomers, the highest scored output structure was used as an input in the Conformational Search panel in Maestro[10]. Thereat, we selected the OPLS3 force field since it previously showed to deliver reliable results in the determination of ligand conformations[11]. We chose the Mixed torsional/Low-mode sampling algorithm with enhanced torsional sampling and a maximal number of 5000 Monte Carlo steps. The conformational search was carried out with water as solvent. For minimization after the conformational search, we selected the Truncated Newton Conjugate Gradient (TNCG) method with the maximal number of iterations set to 500. We left the default convergence threshold of 0.05. The highest ranked structures were selected for the following simulations.

Further, we retrieved the 2D structures of 323 CYP2D6 ligands from the PubChem structure database[7], according to the list published by Rendic and colleagues[6]. We used the cxcalc module provided by ChemAxon[12] to calculate the log D values at physiological pH for all the structures.

Table S2. Ligands used in this study, their structures, abbreviations, and PubChem ID codes.

Ligand	Structure	Abbreviation	PubChem ID code
acetaminophen	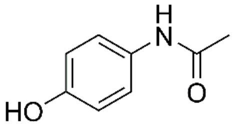	APAP	1983
1,3-butadiene	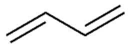	BTD	7845
chlorzoxazone	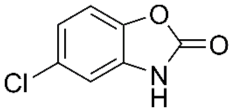	CZX	2733
debrisoquine	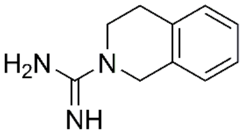	DEB	2966
propofol	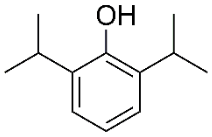	PPF	4943

The structures were created in ChemDraw[13].

Ligand access simulations and validation

The preparation of the protein structures of CYP2D6 as well as the placement of the membrane for the simulations in this study is extensively described elsewhere[2]. In brief, we used a covalently linked combination of the globular domain of CYP2D6 and its corresponding membrane anchor, both preequilibrated in the membrane environment, as a starting point for this study. The globular domain of the protein originally derived from a crystal structure (PDB ID code 3TDA). For simulations with the allelic variant CYP2D6*53, we introduced the mutations according to the PharmVar database[14] in the Maestro graphical user interface (GUI). In 22 simulations, 20 ligands were randomly distributed around the enzyme in the aqueous phase. Additionally, we performed two exploratory simulations with two or six ligands respectively (Table S3). The ligands were randomly translated and rotated relative to the simulation system to obtain unique starting positions. Two exceptions are as follows: simulation #4 was a replica simulation based on simulation #3 started from frame 3500 and simulation #5 was conducted at a different temperature, ensuring that the trajectories were set for a unique course. As the membrane constituent, we chose 1-palmitoyl-2-oleoylphosphatidylcholine (POPC) molecules and built a simulation system using the Desmond System Builder. Next, we used the Desmond Minimization routine to relax the system with 10000 as a maximal number of steps and a convergence threshold of 0.5 kcal/mol/Å^3. Initially, we performed three simulations for each of the five ligands, not only with the aim to study ligand binding, but also to investigate the partitioning of different ligands in a complex system consisting of protein, membrane, and solvent. After observing two access events of BTD and partial access of APAP molecules, we continued (with all settings of the prior simulation being retained) simulations with APAP and launched additional ones to study ligand binding. Simulations were terminated after a binding event or, if no (partial) binding event was taking place, terminated after simulation times between 1.08 µs (simulation #24) and 1.92 µs (simulation #10) for the longest simulation. For the two initial simulations, the temperature was left at the default value of 300.00 K (26.85 °C or 80.33 °F), while we selected the temperature to be either 310.00 K (36.85 °C or 98.33 °F) or 313.15 K (40 °C or 104 °F) for the following simulations. The increased temperature compared to the physiological state represents a patient with fever which is one of the main indications for the pharmacotherapy with APAP[15].

We determined the RMSD as well as the RMSF of the simulations using the Simulation Interaction Diagram panel within Maestro. For these calculations, the residues forming the flexible membrane anchor (residue numbers 1-31) were excluded due to their large movements compared to the rest of the protein. To assess the heme tilt angle during our simulations, we used an in-house script looping over MD frames in the PDB format extracted every 960 ps of the simulations. The heme tilt angle is defined as the angle between the heme plane, defined by the porphyrin nitrogen atoms, and the z-axis of the system representing the membrane normal. Likewise, we used the same frames to calculate the burying depth of the enzyme in the membrane according to the method established by Ducassou and colleagues[16], who defined the distances between the mass centers of the protein α-carbons and the C1 atoms of the membrane molecules as burying depth. As before, we used an in-house routine to pipeline the MD frames through this calculation and determine average values.

The contacts between the ligands were also determined using an in-house python routine that evaluated every frame of the respective MD simulation. Thereat, we determined the number of frames, in which the 5 Å zone around accessing ligand molecule included a heavy atom of another ligand based on individual MD frames exported from the Maestro GUI. We divided the results into three phases according to the progress of the access event.

Residues involved in the translocation of the ligand were determined using the Simulation Interaction Diagram panel in Maestro. Simultaneously, the torsion angles of the ligands were monitored. The adaptation of secondary structure elements was determined based on the RMSD and RMSF diagrams as well as the careful visual examination of the MD trajectories.

A ligand was considered to be in a pose which would allow oxidation reaction to proceed at a site of metabolism (SOM) when the distance between the SOM and the heme iron was between 5 and 7 Å.

Table S3. Simulations performed in this study.

Simulation	Structure	Ligands	Temperature (K)	Duration (μs)
#1	CYP2D6 WT	2x CZX	300.00	1.00
#2	CYP2D6 WT	6x BTD	300.00	1.44
#3	CYP2D6*53	20x APAP	313.15	0.96
#4	CYP2D6*53	20x APAP	313.15	0.70
#5	CYP2D6*53	20x APAP	310.00	1.28
#6	CYP2D6*53	20x APAP	313.15	1.20
#7	CYP2D6*53	20x APAP	310.00	1.40
#8	CYP2D6 WT	20x APAP	313.15	1.44
#9	CYP2D6 WT	20x APAP	313.15	1.92
#10	CYP2D6*53	20x APAP	313.15	1.92
#11	CYP2D6 WT	20x APAP	310.00	0.72
#12	CYP2D6*53	20x BTD	310.00	0.48
#13	CYP2D6*53	20x BTD	310.00	0.48
#14	CYP2D6*53	20x BTD	310.00	0.48
#15	CYP2D6*53	20x CZX	310.00	0.48
#16	CYP2D6*53	20x CZX	310.00	0.48
#17	CYP2D6*53	20x CZX	310.00	0.48
#18	CYP2D6*53	20x DEB	310.00	0.48
#19	CYP2D6*53	20x DEB	310.00	0.48
#20	CYP2D6*53	20x DEB	310.00	0.48
#21	CYP2D6*53	20x PPF	310.00	0.48
#22	CYP2D6*53	20x PPF	310.00	0.48
#23	CYP2D6*53	20x PPF	310.00	0.48
#24	CYP2D6*53	20x APAP	313.15	1.08

Overview of all simulations conducted throughout this study. The used protein structure, the type and number of ligands within the simulation system, the applied temperature, and the total simulation time are shown.

Preference of ligands for protein, tunnels, and membrane

To determine hotspots of ligands on the protein surface, we developed a python script detecting the presence of ligand heavy atoms in the vicinity of the respective amino acid in a range of 5 Å. For glycine, we used the α-carbon atom, while we chose the β-carbon atom for the remaining amino acids. For this calculation we used superimposed frames collected every 960 ps of the corresponding simulation. For APAP, simulations #3 to #11 were included, while simulations #13 and #14 were considered for BTD. The data was averaged for APAP and BTD and visualized on the surface of CYP2D6. The occupancy of the H1 site was determined for the phase of tunnel passage.

Further, we divided the simulation box into three logical compartments to measure the preference of all ligands in the system for any of them. The first compartment consisted of the space not covered by the membrane (denoted as S), while the other two zones divided the membrane into head groups (H) and membrane core (M). We defined the head group region to be located between the mass center of the nitrogen atoms and the mass center of the C2 atoms the POPC molecules. Accordingly, we defined the membrane core to be located between the C2 atoms of the upper and lower POPC leaflets. An in-house python routine determined the location of the mass center of the ligand in z-direction and compared it to the boundaries of the three mentioned compartments. This analysis was performed for simulations #3 to #7 and #11 to #23. To normalize the compared time spans of the simulations, the interval between 200 and 480 ns of each simulation was considered for the analysis with frames being collected every 528 ps. Average values were calculated for every ligand. Since the included simulations of APAP were conducted at two different temperatures, the average results for APAP were divided in two groups (denoted as APAP-1 and APAP-2).

Tunnel analysis

We used CAVER 3.0 to detect and characterize the tunnels in all simulations with a successful access event[17]. For that, we collected MD frames every 960 ps of the simulations, aligned them in the Protein Structure Alignment panel in Maestro, and determined the starting point for the tunnel computation using CAVER Analyst 1.0[18]. We defined the starting point based on the residues E216, D301, and the heme for every simulation. We used a clustering threshold of 4.5, as it was determined to deliver good results in a previous study[2], while the rest of the settings were left on default. The nomenclature of the enzyme tunnels was adapted from Cojocaru and colleagues[19]. Average bottleneck radii, time-evolved bottleneck radii, and bottleneck residues were derived from the output of the tunnel computation.

Ligand-protein and ligand-membrane energies

In the case of a successful access event, we determined the energy between the ligand and the protein with an in-house routine programmed in C++ language in MD frames in the MacroModel file format extracted at an interval of 480 ps over the course of the whole simulation. Interaction energies were calculated with a 12 Å cutoff from the ligand.

$$E_{ele} = \left(1 - \left(\frac{r}{12}\right)^2\right)^2 \frac{Q_1 Q_2}{4\pi\varepsilon_0 r} \quad (I) \qquad E_{H-bond} = \left(\frac{C}{r_{ij}^{12}} - \frac{D}{r_{ij}^{10}}\right) \cdot cos^2(\theta_{Don-H\cdots Acc}) \quad (II) \qquad E_{hydrophobic} = \sum_{ij} E_{hydrophobic}^{ij} \quad (III)$$

$$E_{hydrophobic}^{ij} = \begin{cases} 0.0 \ \ (1 \leq scale) \\ 0.25 \cdot scale^3 - 0.75 \cdot scale \\ 0.5 \ \ (-1.0 < scale < 1.0 \\ 1.0 \ \ (scale \ \leq \ -1.0) \end{cases} \quad (IV) \qquad scale = 2.0 \ \cdot \left(r_{ij} - r_i^{vdw} - r_j^{vdw} - 2.0\right)/3.0 \quad (V)$$

We evaluated partial contributions from electrostatics and hydrogen bonds according to the Yeti force field terms (Equation I and II)[20,21]. The term for hydrogen bonds accounts for their directionality. The energies for hydrophobic contributions were calculated according to a term adapted from the VSGB 2.0 model (Equations III-V)[22,23]. Membrane molecules were included in the analysis. Even though enhanced sampling methods would have been suited to derive a free energy surface for the uptake process, the ligands in our setup intensively sampled the unbound and bound state allowing to draw conclusions based on differences between these two states.

Hydration analyses

We used an in-house routine to determine the number of the first-shell water molecules around the ligands in every frame of the simulations with a successful access event. The routine determined the number of water molecules in a given MD frame within a distance of 3.5 Å from any ligand atom.

To determine the degree of active site desolvation in response to ligand binding, we extracted MD frames in a frame step of 480 ps covering a spherical zone of 15 Å around active site residues and the ligand of interest. Thereat, the residues 110, 112, 120, 121, 209, 212, 213, 216, 244, 247, 248, 297, 300, 301, 304, 305, 308, 309, 370, 443, 483, and 484 were included due to their proximity to the co-crystallized ligand in the underlying crystal structure (PDB ID code 3TDA). Frames with no ligand atoms detected in the binding site were included for calculating of the average number of waters in the empty state (unliganded). On the other hand, only frames with all ligand atoms present within the binding site were included for calculating of the average number of waters in the occupied state (liganded).

Docking and pose comparison

The ligands for which an access event could be observed in the MD simulations were docked into the active site of CYP2D6. The 3D ligand structures of APAP and BTD were available from previous steps. Prior to docking, we used MGL Tools (v.1.5.6)[24] to prepare the receptor and ligand structures. We defined the search space to be cubic with a side length of 45 Å, manually changed the charge of the heme iron to Fe^{2+} in the PDBQT file, and removed sodium atoms interfering with the calculation. Due to the high flexibility of CYP enzymes[25], including the potential structural adaptations related to ligand binding[19,26–28], we considered several residues to be flexible for our docking calculations. In particular, we selected 112, 120, 211, 216, 221, 244, 296, 297, 301, 304, and 483 to be flexible. We used two different protein structures derived from simulation #3, differing in the orientation of APAP, as input structures. To enrich the results, docking runs were performed in the presence and absence of structural water molecules. Additionally, we performed docking runs with the amide bond of APAP regarded as flexible and rigid respectively. The docking was performed using AutoDock Vina (v.1.1.2)[29] with an exhaustiveness of 8. After docking, the obtained poses were filtered according to three criteria: (i) site of metabolism (SOM) in known range for metabolic reaction; (ii) ligand located within known binding site region; and (iii) docking score higher than -5.5 kcal/mol for the poses of APAP. In the case of BTD, only one pose derived from docking met the criteria.

To compare the poses from docking with ligand poses obtained from the MD simulations presenting successful ligand access, we chose the highest scored docking pose complying with the above-mentioned criteria to determine its similarity to MD poses. Therefore, we extracted all frames of the respective simulations, in which the ligand occupied the binding site, and aligned them to the corresponding docking pose using the Protein Structure Alignment tab within Maestro. Next, we removed all atoms from these frames except for the ligand of interest to ultimately compare the poses using the rmsd.py script provided by Schrodinger. Hydrogen atoms were excluded to compare the similarity between the poses.

Results and Discussion

Access of CYP2D6 ligands from the protein-membrane interface

Figure S1. Analysis of ligand starting positions based on distance to specific protein atoms.

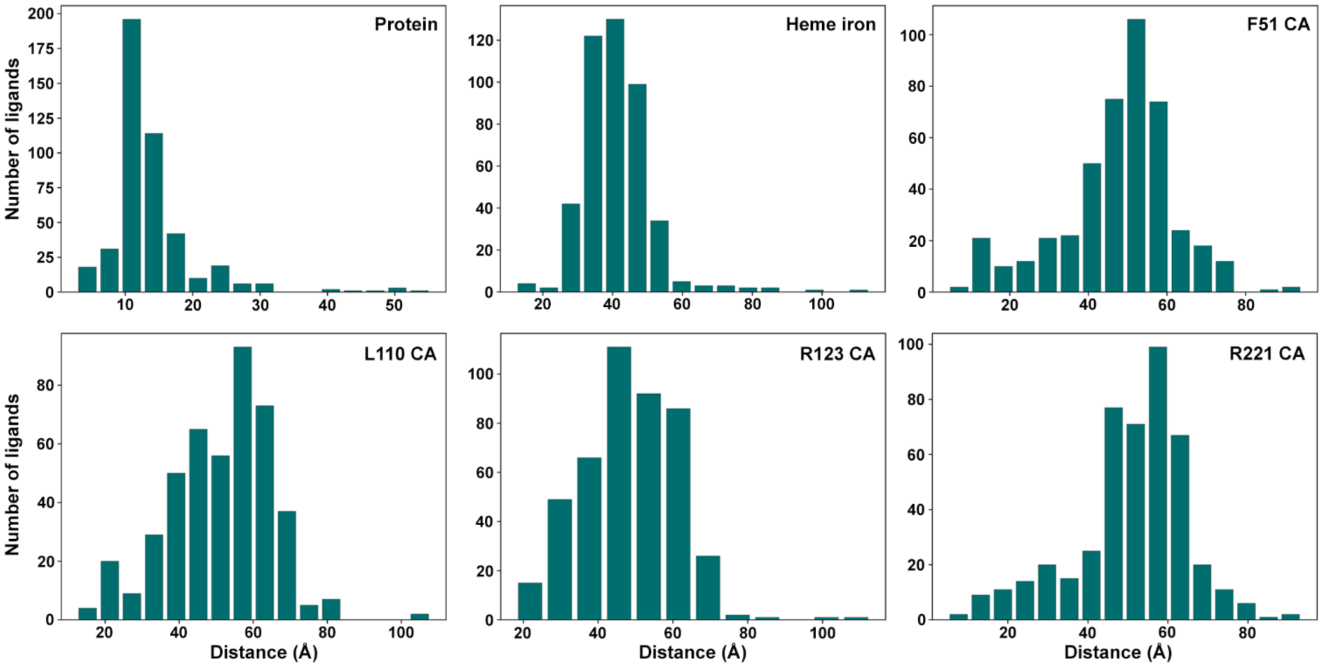

The distances from the ligand starting positions to selected protein atoms are shown. Only heavy atoms were considered. The plots were created in Matplotlib[30].

Figure S2. Location of the tunnels in CYP 2D6

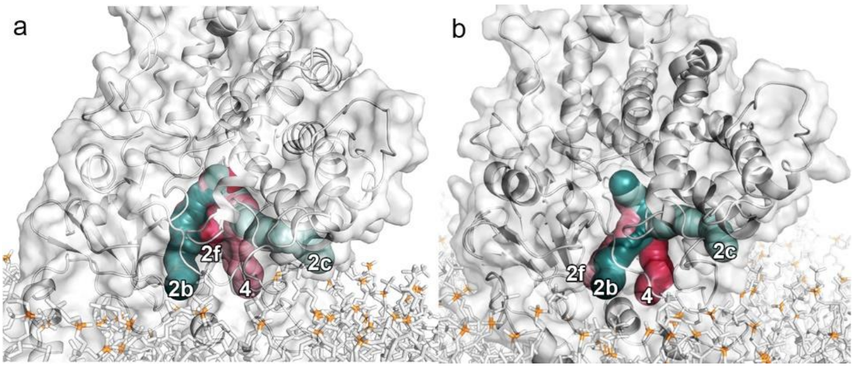

The location of the four most important tunnels in (a) CYP2D6*53 and (b) wild-type CYP2D6.

Table S4. Minimal distances from the starting positions of accessing ligands to protein atoms.

Simulation	Ligand	Ligand-Iron (Å)	Ligand-Protein (Å)
#3	APAP-18	29.1	7.3
#4	APAP-7	84.0	55.3
	APAP-18	18.2	2.9 [a]
#5	APAP-18	29.1 [b]	7.3 [b]
#6	APAP-6	36.2	12.0
#7	APAP-3	55.7	23.8
	APAP-8	46.1	13.7
#8	APAP-20	36.3	13.0
#13	BTD-11	42.4	8.3
#14	BTD-3	35.4	12.4

The minimal distances from the heavy atoms of the accessing ligand molecules to the heme iron and the next protein heavy atom are shown. [a] Since this was a replica simulation, this ligand started near the entrance of tunnel 2f. [b] Simulations #5 was started from the same coordinates as simulation #3 at a different temperature.

Model validation

The root mean square deviation (RMSD) of the protein backbone indicated a good convergence of the systems besides minor drifts (Figure S3a). Except for one simulation, the values mostly remained between 2 and 3 Å. The RMSD diagram of simulation #3 presented several spikes that were caused by the movement of P267 as it was indicated by the high root mean square fluctuation (RMSF) of this particular residue located in the flexible GH loop on the protein surface (Figure S3b). A visual examination of the simulation revealed a reversible contraction of the loop after the ligand reached the active site, explaining the increased RMSD and RMSF values. However, the last frame of the simulation showed a value of 2.9 Å similar to the other simulations confirming convergence. In general, the RMSF diagrams indicated similar regions of local flexibility among the simulations that were in agreement with our previously published data[2]. The burying depth of the globular domain is used to validate and compare membrane-anchored models of CYPs[16]. The averages (Table S5) as well as the time-evolved values (Figure S3c) presented a narrow range around 38.5 Å comparable to the experimentally determined value of 35±9 Å and our previous observations[2,31]. On the other hand, the heme tilt angle, describing the angle between the z-axis and the plane of the porphyrin nitrogens of the heme, showed stronger fluctuations (Table S5 and Figure S3c). Nevertheless, the fluctuations were within the boundaries of 38-78° reported based on rotational diffusion measurements performed in proteoliposomes[32].

The placement of multiple ligands in a simulation system to study rare molecular events, as it was used in previous studies[33,34], comes with advantages as well as disadvantages. Obviously, an advantage is the increased likeliness of observing a ligand accessing the enzyme, while a disadvantage is the potential influence of the ligands on each other. Even though molecules regularly contact each other in the crowded cellular environment[35], the comparably limited size of a simulation box (e.g. 104.6 x 128.5 x 191.5 $Å^3$ in simulation #3) could have potentially intensified such phenomena. Therefore, we determined the degree to which the accessing ligands contacted other ligand molecules during the different phases of the uptake process (Table S6). The percentage of frames, in which a heavy atom of the accessing ligand was within a radius of 5 Å from another heavy atom of a different ligand, was generally low for the BTD molecules. In the case of APAP, the values were scattered between 2.3 and 36.3%. High values were observed in the case of a dual ligand access, where the concurrent occupation of the active site naturally led to contacts between the two ligands. In simulations #5 and #8 however, we observed increased values despite only a single molecule accessing the enzyme. In the case of simulation #5, the contacts were low during the recognition and translocation phases. The high values, when the accessing ligand occupied the active site, were caused by an additional APAP molecule located on the surface of the enzyme in around 4.5 Å distance among their heavy atoms. In simulation #8, the contacts occurred during the recognition phase, when multiple ligand molecules formed transient agglomerates before APAP-20 initiated its translocation to the active site. In summary, the uptake process was not influenced by ligand contacts in our simulations with the exception of dual access events. To check for potential aggregation of the ligand molecules during the simulations, we used an in-house Python routine to determine the average distance of the mass centers among all ligand molecules in each MD frame of simulations #3 to #24.

Table S5. Validation parameter average values.

Simulation	Heme tilt angle (Å)	Burying depth (Å)
#3	57.0 ± 8.5	38.9 ± 2.3
#4	54.8 ± 6.3	38.8 ± 1.8
#5	48.3 ± 8.0	38.1 ± 1.7
#6	57.4 ± 8.0	38.4 ± 1.6
#7	57.0 ± 5.8	38.5 ± 1.6
#8	38.8 ± 7.0	38.0 ± 1.6
#13	44.0 ± 7.0	38.6 ± 1.5
#14	53.6 ± 5.4	39.4 ± 1.4

The values are shown with standard deviation.

Table S6. Contacts of accessing molecules to other ligands.

Simulation	Ligand	Recognition	Translocation	Active site	Total
#3	APAP-18	0<br>(n= 84)	7.8<br>(n= 5771)	0.0<br>(n=14145)	2.3<br>(n= 20000)
#4	APAP-7	3.9<br>(n= 7211)	78.7<br>(n= 3896)	56.1<br>(n= 3481)	36.3<br>(n= 14588)
	APAP-18	n/a[a]	27.1<br>(n= 14588)	n/a[a]	27.1<br>(n= 14588)
#5	APAP-18	0.6<br>(n= 313)	7.7<br>(n= 16792)	35.1<br>(n= 9562)	17.4<br>(n= 26667)
#6	APAP-6	4.5<br>(n= 12731)	0<br>(n= 1583)	0<br>(n= 10689)	2.3<br>(n= 25003)
#7	APAP-3	14.4<br>(n= 5646)	0<br>(n= 3895)	47.5<br>(n= 19627)	34.7<br>(n= 29168)
	APAP-8	5.7<br>(n= 12751)	19.5<br>(n= 8501)	96.9<br>(n= 7914)	34.5<br>(n= 29168)
#8	APAP-20	36.8<br>(n=29573)	0<br>(n=428)	n/a [b]	36.3<br>(n=30001)
#13	BTD-11	1.0<br>(n= 605)	0<br>(n= 583)	0<br>(n= 8814)	0.1<br>(n= 10002)
#14	BTD-3	13.6<br>(n= 22)	0<br>(n= 62)	0<br>(n=7271)	1.1<br>(n= 7355)

The percentages of frames, in which the accessing molecules contacted surrounding ligands is divided into three phases. Contacts between heavy atoms in the range of 5 Å were considered. Together with the percentage, the number of frames in the respective interval is given. [a] Since simulation #4 was a replica starting in the entrance of tunnel 2f, but did not reach the active site in a conformation that would allow a metabolic reaction. [b] The ligand molecule did not reach a conformation in agreement with a metabolic reaction.

Figure S3. Validation of simulations with a successful access event.

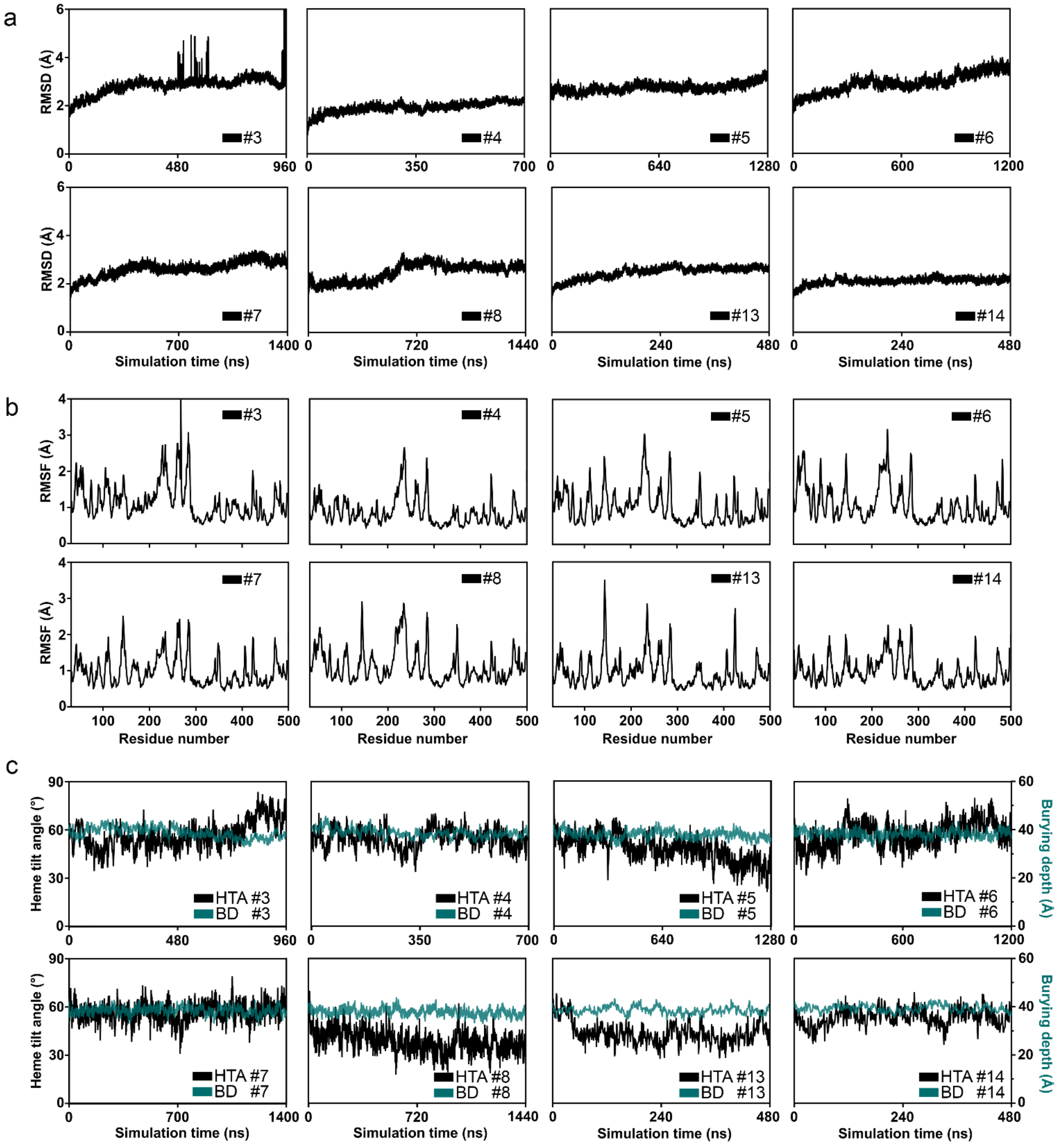

(a) The RMSD of all eight simulations presenting a successful access event is shown. The corresponding simulation identifier is indicated at the bottom right of the plots. (b) The RMSF of the simulations presenting a successful access event is shown. The corresponding simulation identifier is indicated at the top right of the plots. (c) The heme tilt angle (HTA) for simulations presenting a successful access event is shown together with the burying depth (BD) of the enzyme. The heme tilt angle is shown in black, while the burying depth is colored pine green.

Figure S4. Distances between all ligand in the simulations as a check for aggregation.

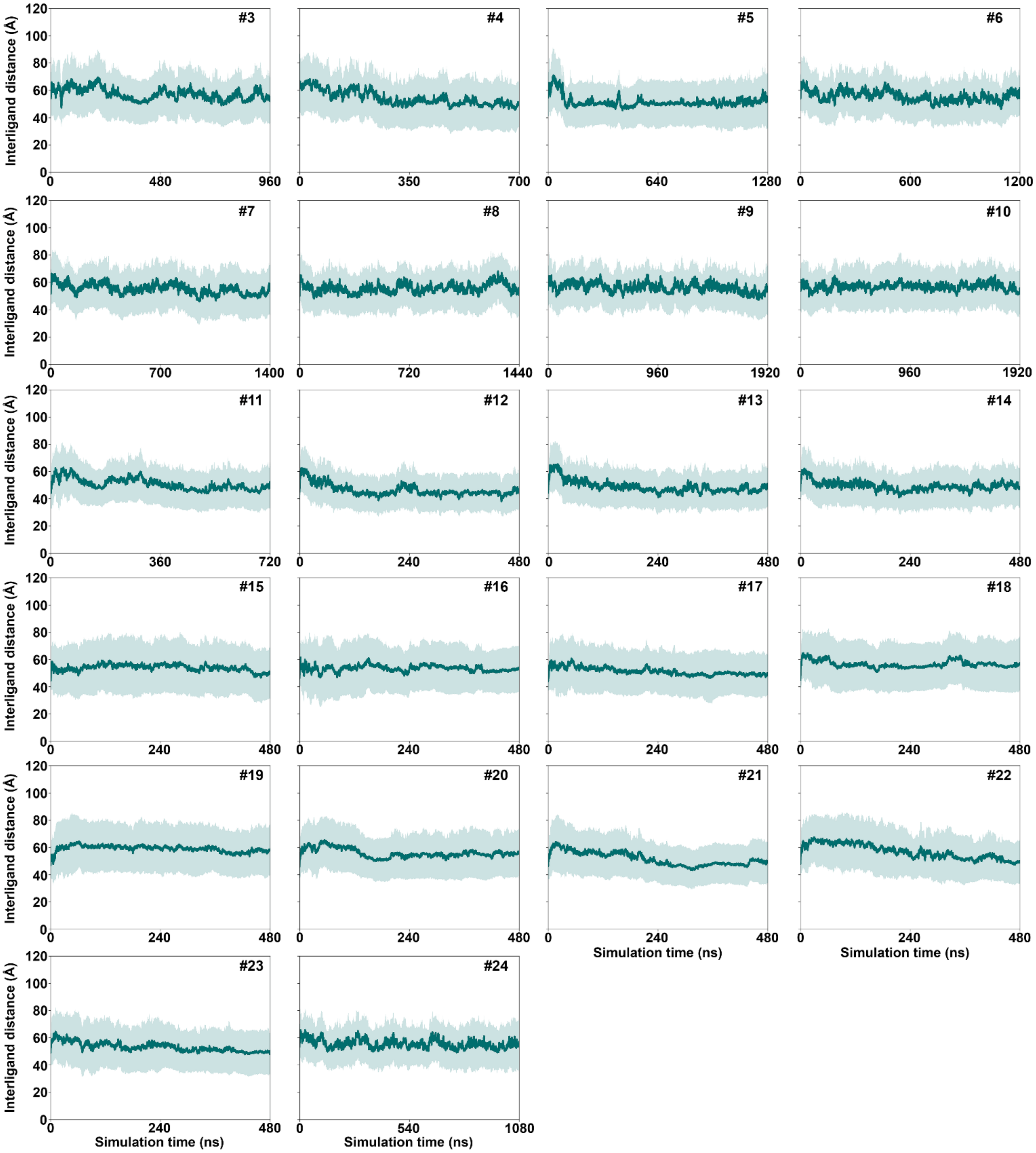

The distances between the mass centers of all ligands within a single MD frame were measured and averaged for simulations #3 to #24. This analysis has been performed as a check for aggregation.

Preference of ligands for the protein, tunnels, and the membrane

Table S7. Occupancy of H1 allosteric site during ligand access.

Simulation	Ligand	Tunnel	Occupancy
#3	APAP-18	2f	yes
#4	APAP-7	2f	yes
	APAP-18	2f	yes
#5	APAP-18	4	no
#6	APAP-6	2b	no
#7	APAP-3	2b	no
	APAP-8	2b	no
#8	APAP-20	2f	yes
#13	BTD-11	2c	no
#14	BTD-3	2c	no

The determined occupancy of the potential allosteric site H1 is shown together with the simulation identifier, the accessing ligand and the preferred tunnel.

Figure S5. Ligand hotspots on the protein surface.

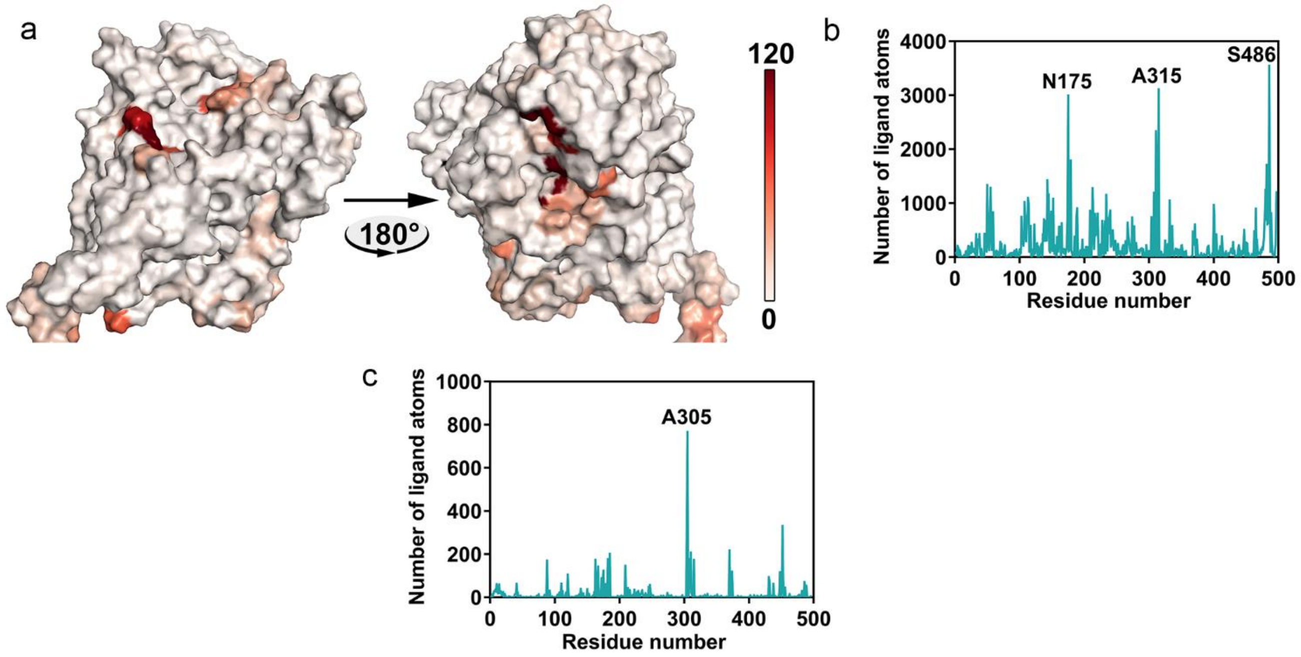

(a) The visualization of ligand hotspots on the surface of CYP2D6 determined for BTD is shown. The scale from 0-120 describes the cumulative number of ligand heavy atoms in a 5 Å radius of the CB atom (CA atom for glycine) of the protein amino acids. (b) A plot of the ligand hotspots of APAP for CYP2D6. The comparably intensive peaks of N175, A315, and S486 are indicated. (c) A plot of the ligand hotspots of BTD for CYP2D6. The comparably intensive peak of A305 is indicated.

Structural adaptation of the protein

Table S8. Adaptation of the secondary structure of CYP2D6.

Simulation	Residues	Secondary structure	Ligand involvement
#3	V49-F58	αA' and αA	directly
#4	H48-L61	αA' and αA	directly
#5	V229-L236	FG loop	directly
	S288-N291	HI loop	distant
#6	H48-L61	αA' and αA	distant
#7	n/a	n/a	n/a
#8	n/a	n/a	n/a
#13	L110-S116	BC loop	directly
	K283-K288	HI loop	distant
#14	E280-S289	BC loop and αB	distant

The structural adaptations of the protein secondary structure during ligand access are shown. The involvement of the ligand was visually determined and was classified to be either directly or distant. For simulations #7 and #8, no adaptations were observed.

Figure S6. Most prominent bottleneck residues in CYP2D6.

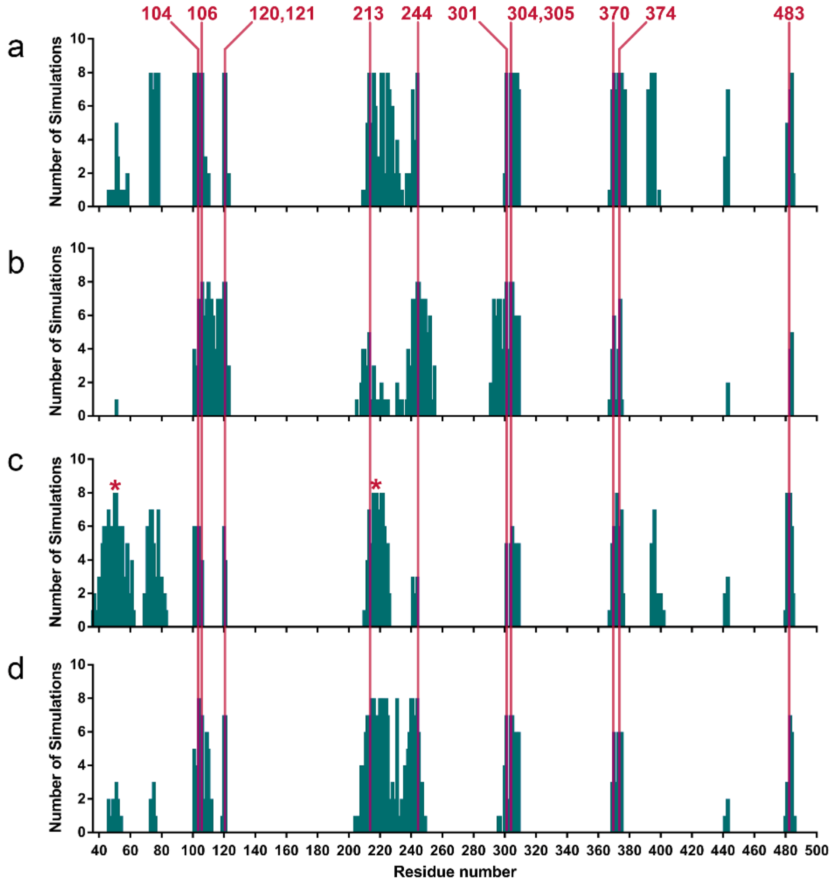

The major bottleneck residues for all simulations presenting a successful access event are shown with the number of simulations, in which the residues participated in bottlenecking the respective tunnel. Residues with high scores are indicated, while the gating residues F51 and F219 are pinpointed by red asterisks. The results are shown for (a) tunnel 2b, (b) tunnel 2c, (c) tunnel 2f, and (d) tunnel 4.

Figure S7. Bottleneck radii of enzyme tunnels.

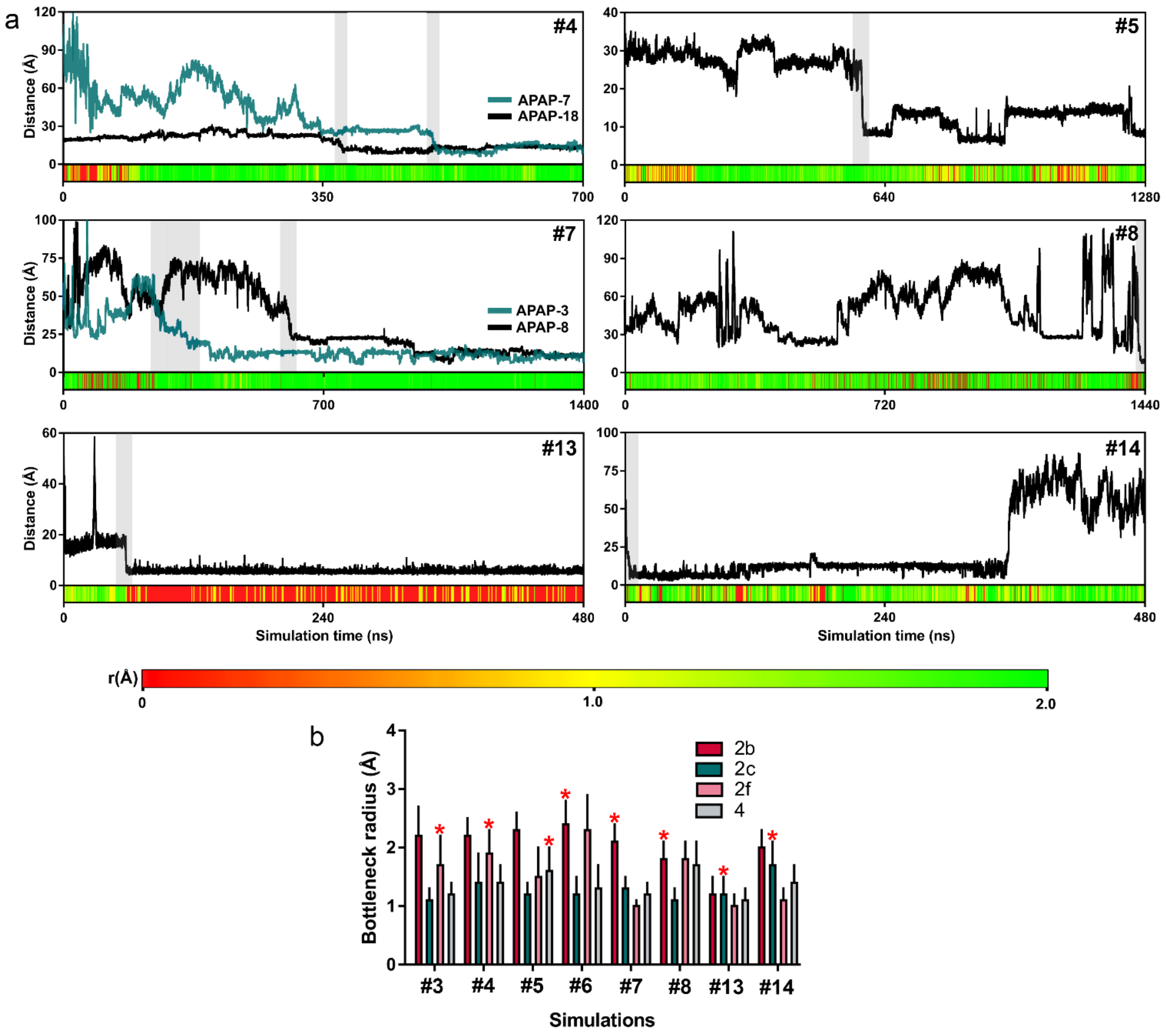

(a) The distance of the ligand SOM is plotted against the simulation time and the time-evolved bottleneck radius. The simulation identifiers are shown at the top right of the plots. Gray bars indicate the period of tunnel passage. The legend below indicates the coloring scheme for the bottleneck radii. (b) The average bottleneck radii for the simulations presenting a successful access event are shown. The tunnels, which were used by the ligand in the respective simulation are indicated by red asterisks. The values are shown with standard deviation.

Table S9. Characteristics of docking poses meeting the selection criteria.

Pose	Ligand	Protein	Score (kcal/mol)	Ligand flexibility	Water
A1	APAP	a	-8.1	rigid	yes
A2	APAP	a	-7.4	flexible	yes
A3	APAP	a	-6.8	flexible	no
A4	APAP	a	-6.7	rigid	no
A5	APAP	a	-6.8	flexible	yes
A6	APAP	a	-6.7	rigid	no
A7	APAP	b	-6.7	flexible	yes
A8	APAP	a	-6.5	rigid	yes
A9	APAP	a	-6.3	rigid	yes
A10	APAP	a	-6.2	rigid	no
B1	BTD	a	-4.4	n/a	yes

Overview of the predicted binding free energies (Score) for docking poses obtained for APAP and BTD with two different protein structures and different parameters regarding ligand flexibility and the presence of water molecules. Only poses matching the inclusion criteria (see SI Computational Methods) are shown. Note that only one pose of the docking calculations with BTD fulfilled the selection criteria and no special restraints were applied to the bonds of BTD.

Table S10. Comparison of poses from docking and MD simulations.

Simulation	Ligand	RMSD (Å)
#3	APAP-18	0.28
#4	APAP-7	4.96
	APAP-18	4.60
#5	APAP-18	3.57
#6	APAP-6	0.42
#7	APAP-3	4.49
	APAP-8	5.93
#8	APAP-20	4.07
#13	BTD-11	2.89
#14	BTD-3	1.79

The heavy atom RMSD between the selected docking pose and the closest resembling pose from MD simulations is shown.

The driving forces for translocation

During the binding process to an active site, ligands generally experience some degree of strain[36] associated with a penalty toward binding affinity on the target. Our results did not reveal a clear trend for a reduced conformational freedom inside the enzyme based on the ligand torsion angles (Fig. S10). Especially for BTD, such a result was to be expected since it is not able to fill the volume of enzyme tunnels or the active site to a similar degree as APAP due to its smaller size. In this case, our calculations were hampered by hardly comparable time intervals. Even when the number of frames inside and outside the enzyme were similar (Figure S8 D, G, I), the results were still inconclusive. While APAP-8 in simulation #7 showed a clear restriction in its conformational freedom inside the enzyme despite a higher number of frames for this period, the other ligands did not behave similarly. Frequently, the diversity of visited torsion angle values was rather altered than restricted, indicating a limited constraint on the ligand. Furthermore, the number of torsions in APAP and BTD is limited, potentially reducing the impact of ligand strain compared to a larger molecule such as the cocrystallized ligand prinomastat.

The number of water molecules measured in the active site cavity was similar to observations other CYPs[37].

Figure S8. Energetic contributions from electrostatics and hydrogen bonds between the ligand and the protein.

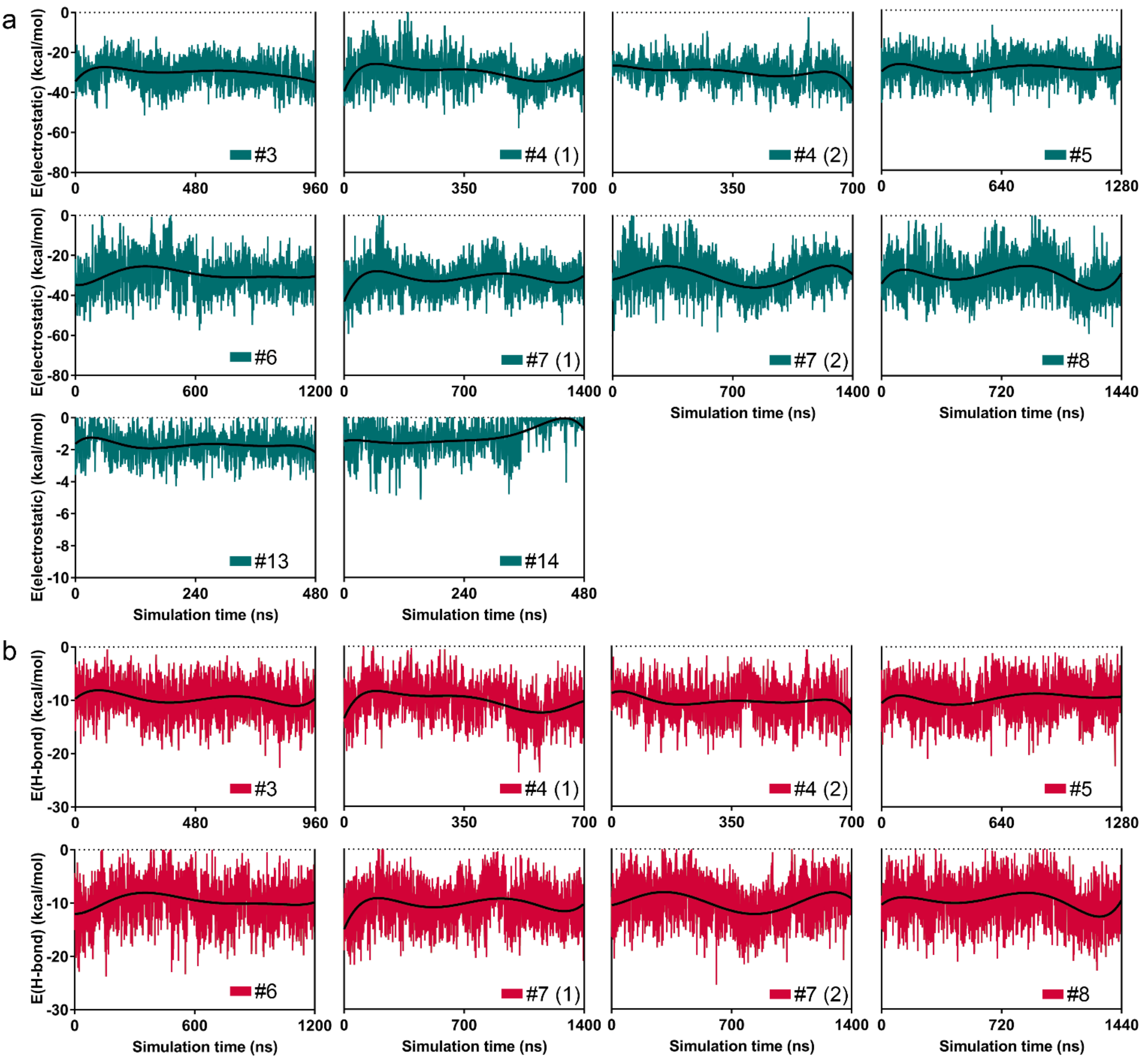

The energetic contributions of (a) electrostatics and (b) hydrogen bonds in simulations presenting a successful access event are shown. The simulation identifier is indicated at the bottom right. The regression lines show the centered sixth order polynomial fitted to the values. In the case of a double access event, the results were numbered sequentially after Table 1 in the main article.

Figure S9. Polar interactions and hydration shell of the ligands.

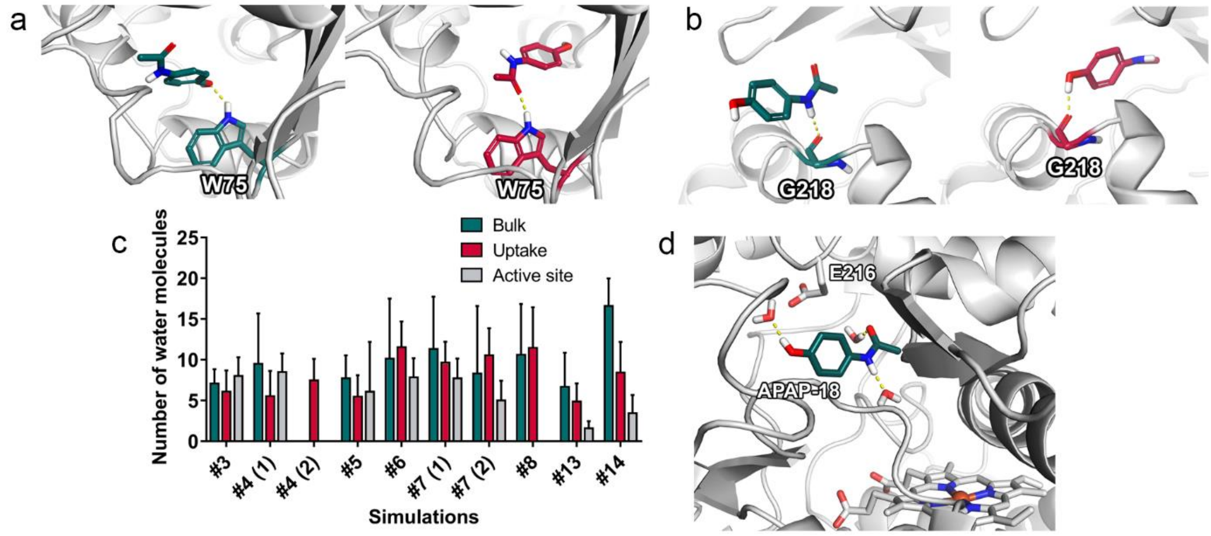

(a) The interaction between APAP and W75 shown at two different time points of simulation #4. (b) The interaction between APAP and G218 in simulation #4 shown at two different time points. (c) The number of water molecules in a 3.5 Å radius around the ligand are shown for the three phases of ligand uptake. In the case of a double access event, the data is sequentially shown for the ligands according to the order in Table 1. The values are presented with standard deviation. (d) The ligand is shown surrounded by water molecules in simulation #3. Note the hydrogen bonds between the ligand and the water molecules. For orientation, the location of E216 is shown at the top of the figure.

Table S11. Residues interacting with the ligand during the access process.

Simulation	Tunnel	Residues recognition	Residues translocation	Residues active site
#3	2f	F51, L46, V49, G218, F219	V49, F51, L73, W75, F219, V370, L372, T375, T394	R101, G367, I369, V370, V374, T375, F483
#4 (1)	2f	N45, L46, V49, D50, F51, F219	V49, F51, Q52, T54, L73, W75, L213, E216, G218, R221, E222, V370, T375	R101, L213, E216, S304, P371, V370, V374, F483
#4 (2)	2f	N45, L46, V49, F51, T54, L73, S217, G218, L372	L46, V49, F51, L73, W75, G218, F219, F243, F247, S304	n/a
#5	4	E215, L220, R221, R242, K245	E216, L224, N225, V227, Q244, K245, F247, T375, F483	E216, F247, S304, F483
#6	2b	R25, R26, R123, K391	H48, E216, E222, V370, T375, T394, F481	I120, E216, S304, V370, V374, T375, F483
#7 (1)	2b	Q108, N225, L231, H232	F51, T54, W75, P103, Q108, A209, E216, R221, E222, G373, T394, F483	F247, D301, S304, T375
#7 (2)	2b	R101, F112, Q117, L121, R123	F51, P103, R123, E216, E222, N225, E244, F247, D301, T394, F483	E216, F247, D301, S304, F483
#8	2f	Q52, N53, K214, F481, A482	F120, L213, E216, A305, A308, V370, T375	n/a
#13	2c	L110, F112	L110, F112, I120, L121, I297	I120, L213, A305, V370, V374
#14	2c	I106, I109, L110, L241	L121, L241, A305, F483, L484	I120, L213, F243, F247, A305, V370, V374

The residues that were determined to interact with the respective ligand are shown for the corresponding phase of the access event. APAP-18 in simulation #4 as well as APAP-20 in simulation #8 did not adopt pose in the active site that is in accordance with a metabolic reaction. In the case of a double access event, the data is sequentially shown for the ligands according to the order in Table 1.

Figure S10. Distribution of torsion angle values of the accessing ligands inside and outside the enzyme.

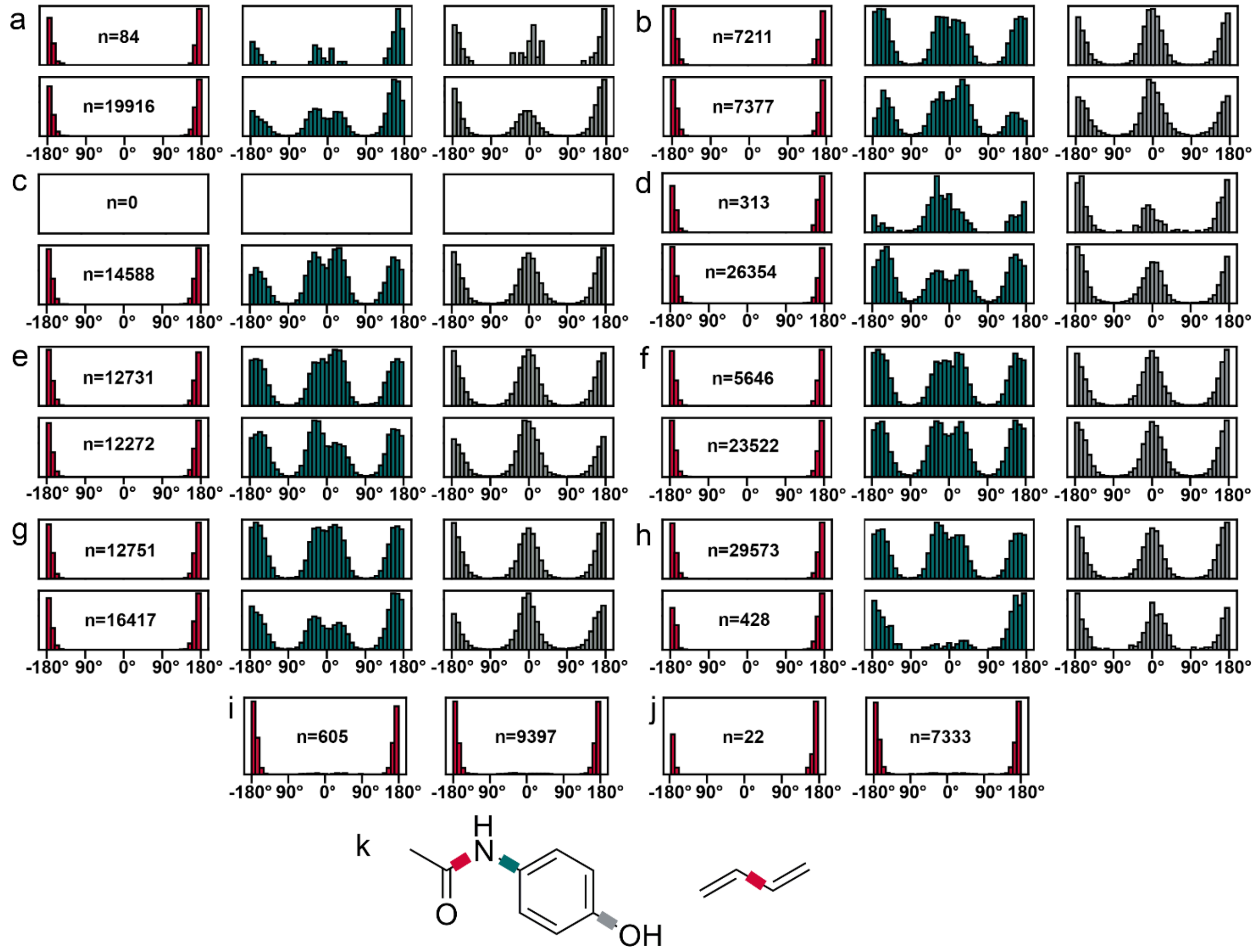

(a) For all simulations the values outside the enzyme (top) are compared with the ones where the ligand was inside the enzyme (bottom). The number of frames for the respective interval are described by n. The plots were generated using Matplotlib[30]. Here, the torsion angles of APAP-18 in simulation #3 are shown. (b) Torsion angles of APAP-7 in simulation #4. (c) Torsion angles of APAP-18 in simulation #4. (d) Torsion angles of APAP-18 in simulation #5. (e) Torsion angles of APAP-6 in simulation #6. f) Torsion angles of APAP-3 in simulation #7. (g) Torsion angles of APAP-8 in simulation #7. (h) Torsion angle of APAP-20 in simulation #8. (i) Torsion angles of BTD-11 in simulation #13. (j) Torsion angles of BTD-3 in simulation #14. (k) The torsion angles of both ligands are shown with the corresponding color used to plot. While APAP is shown on the left side, BTD is shown on the right side. The molecular structures were created in ChemDraw[13].

Supporting Movie

Movie S1. Access events.
The movie shows the access events of both APAP and BTD to the active site of CYP2D6. At the beginning of the movie, the complete simulation system is shown for improved comprehension. The individual clips were cut, after the ligand(s) reached the binding site. The video will be made available for download on our GitHub repository at https://github.com/mmodbasel/scripts-001.

References

1. Bowers, K. *et al.* Scalable Algorithms for Molecular Dynamics Simulations on Commodity Clusters. *ACM/IEEE SC 2006 Conf.* 43–43 (2006). doi:10.1109/SC.2006.54
2. Fischer, A., Don, C. G. & Smieško, M. Molecular Dynamics Simulations Reveal Structural Differences among Allelic Variants of Membrane-Anchored Cytochrome P450 2D6. *J. Chem. Inf. Model.* **58**, 1962–1975 (2018).
3. Shaw, D. E. *et al.* Anton 2: Raising the Bar for Performance and Programmability in a Special-Purpose Molecular Dynamics Supercomputer. *Int. Conf. High Perform. Comput. Networking, Storage Anal. SC* **2015–Janua**, 41–53 (2014).
4. Schrodinger LLC. The PyMOL Molecular Graphics System, Version 2.1.1. (2017).
5. Graphpad Software. GraphPad Prism version 7.00. (2016).
6. Rendic, S. Summary of information on human CYP enzymes: Human P450 metabolism data. *Drug Metab. Rev.* **34**, 83–448 (2002).
7. Kim, S. *et al.* PubChem Substance and Compound databases. *Nucleic Acids Res.* **44**, D1202-13 (2016).
8. Small-Molecule Drug Discovery Suite 2017-4, Schrödinger, LLC, New York, NY, 2017.
9. Greenwood, J. R., Calkins, D., Sullivan, A. P. & Shelley, J. C. Towards the comprehensive, rapid, and accurate prediction of the favorable tautomeric states of drug-like molecules in aqueous solution. *J. Comput. Aided. Mol. Des.* **24**, 591–604 (2010).
10. Small-Molecule Drug Discovery Suite 2017-4, Schrödinger, LLC, New York, NY, 2017.
11. Gürsoy, O. & Smieško, M. Searching for bioactive conformations of drug-like ligands with current force fields: How good are we? *J. Cheminform.* **9**, 1–13 (2017).
12. Marvin 17.27.0, ChemAxon (http://www.chemaxon.com), 2017.
13. ChemDraw Professional 16.0.1.4, PerkinElmer Informatics, 2017.
14. Gaedigk, A. *et al.* The Pharmacogene Variation (PharmVar) Consortium: Incorporation of the Human Cytochrome P450 (CYP) Allele Nomenclature Database. *Clin. Pharmacol. Ther.* **0**, 4–6 (2017).
15. McGuinness, S. *et al.* Acetaminophen for Fever in Critically Ill Patients with Suspected Infection. *N. Engl. J. Med.* **373**, 2215–2224 (2015).
16. Ducassou, L. *et al.* Membrane-bound human orphan cytochrome P450 2U1: Sequence singularities, construction of a full 3D model, and substrate docking. *Biochimie* **140**, 166–175 (2017).
17. Chovancova, E. *et al.* CAVER 3.0: A Tool for the Analysis of Transport Pathways in Dynamic Protein Structures. *PLoS Comput. Biol.* **8**, 23–30 (2012).
18. Kozlikova, B. *et al.* CAVER Analyst 1.0: Graphic tool for interactive visualization and analysis of tunnels and channels in protein structures. *Bioinformatics* **30**, 2684–2685 (2014).
19. Cojocaru, V., Winn, P. J. & Wade, R. C. The ins and outs of cytochrome P450s. *Biochim. Biophys. Acta - Gen. Subj.* **1770**, 390–401 (2007).
20. Vedani, A., Dobler, M., Hu, Z. & Smieško, M. OpenVirtualToxLab-A platform for generating and exchanging in silico toxicity data. *Toxicol. Lett.* **232**, 519–532 (2015).
21. Vedani, A. & Huhta, D. W. A new force field for modeling metalloproteins. *J. Am. Chem. Soc.* **112**, 4759–4767 (1990).
22. Li, J. *et al.* The VSGB 2.0 model: A next generation energy model for high resolution protein structure modeling. *Proteins Struct. Funct. Bioinforma.* **79**, 2794–2812 (2011).
23. Verdonk, M. L., Cole, J. C., Hartshorn, M. J., Murray, C. W. & Taylor, R. D. Improved protein–ligand docking using GOLD. *Proteins Struct. Funct. Bioinforma.* **52**, 609–623 (2003).
24. Morris, G. & Huey, R. AutoDock4 and AutoDockTools4: Automated docking with selective receptor flexibility. *J Comput Chem.* **30**, 2785–2791 (2009).
25. Hendrychová, T. *et al.* Flexibility of human cytochrome P450 enzymes: Molecular dynamics and spectroscopy reveal important function-related variations. *Biochim. Biophys. Acta - Proteins Proteomics* **1814**, 58–68 (2011).
26. Poulos, T. L. Cytochrome P450 flexibility. *Proc. Natl. Acad. Sci.* **100**, 13121–13122 (2003).
27. Winn, P. J., Lüdemann, S. K., Gauges, R., Lounnas, V. & Wade, R. C. Comparison of the dynamics of substrate access channels in three cytochrome P450s reveals different opening mechanisms and a novel functional role for a buried arginine. *Proc. Natl. Acad. Sci. U. S. A.* **99**, 5361–5366 (2002).
28. Gora, A., Brezovsky, J. & Damborsky, J. Gates of enzymes. *Chem. Rev.* **113**, 5871–5923 (2013).
29. Brooks, B. R. *et al.* AutoDock Vina: Improving the Speed and Accuracy of Docking with a New Scoring Function, Efficient Optimization, and Multithreading. *J. Comput. Chem.* **30**, 1545–1614 (2009).
30. Hunter, J. D. Matplotlib: A 2D Graphics Environment. *Comput. Sci. Eng.* **9**, 90–95 (2007).
31. Bayburt, T. H. & Sligar, S. G. Single-molecule height measurements on microsomal cytochrome P450 in nanometer-scale phospholipid bilayer disks. *Proc. Natl. Acad. Sci.* **99**, 6725–6730 (2002).
32. Ohta, Y., Kawato, S., Tagashira, H., Takemori, S. & Kominami, S. Dynamic Structures of Adrenocortical Cytochrome P-450 in Proteoliposomes and Microsomes: Protein Rotation Study. *Biochemistry* **31**, 12680–12687 (1992).
33. Shan, Y. *et al.* How does a drug molecule find its target binding site? *J. Am. Chem. Soc.* **133**, 9181–9183 (2011).
34. Dror, R. O. *et al.* Pathway and mechanism of drug binding to G-protein-coupled receptors. *Proc. Natl. Acad. Sci.* **108**, 13118–13123 (2011).
35. Smith, S., Cianci, C. & Grima, R. Macromolecular crowding directs the motion of small molecules inside cells. *J. R. Soc. Interface* **14**, 20170047 (2017).
36. Mobley, D. L. & Dill, K. A. Binding of small-molecule ligands to proteins: 'what you see' is not always 'what you get'. *Structure* **17**, 489–498 (2009).
37. Rydberg, P., Rod, T. H., Olsen, L. & Ryde, U. Dynamics of water molecules in the active-site cavity of human cytochromes P450. *J. Phys. Chem. B* **111**, 5445–5457 (2007).

Author Contributions

Author contributions: A.F. and M.S. designed the research, A.F. performed the research, A.F. and M.S. wrote the paper, M.S. administrated the project. A.F. is lead author, M.S. is corresponding author.
